# Supplementary material for: NSAID-Induced Enteropathy Affects Regulation of Hepatic Glucose Production by Decreasing GLP-1 Secretion
Source: Nutrients. 2021 Dec 28;14(1):120. doi: 10.3390/nu14010120 (PMC8746549; doi:10.3390/nu14010120)
Supplement: Supplementary file 1 [file nutrients-14-00120-s001.zip › nutrients-1509621-supplementary.pdf]

Supplementary materials

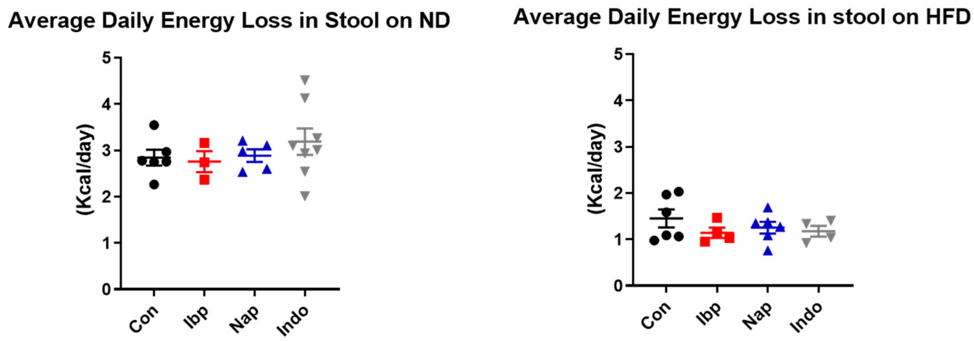

Figure S1. Prolonged treatment with variable NSAIDs over 6 weeks has no effect on calorie absorption. Average daily energy loss in stool expressed in (Kcal/day) after 6 weeks of oral gavage with PBS (Con), ibuprofen (Ibp), naproxen, (Nap), or indomethacin (Indo) in C57 Blc/6J male mice placed on either normal diet (ND) or high-fat diet (HFD). All results are presented as mean ± SEM (error bars). Student's t test was used to compared means of each NSAID group to the control group. *n* = 6 (Con), 4 (Ibp), 5-6 (Nap), and 4-7 (Indo).

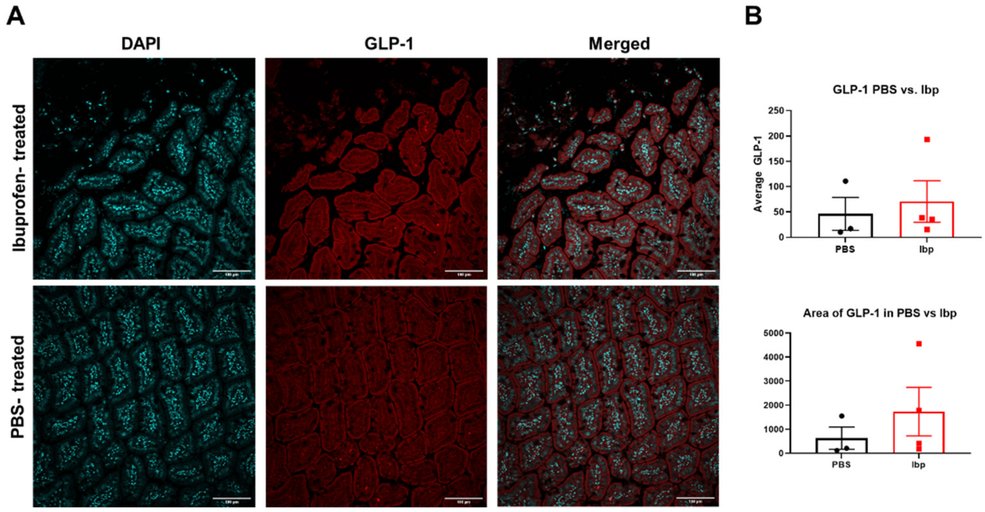

Figure S2. Representative field of view of GLP-1 Immunofluorescence staining in mice ileum. (**A**) Confocal images of GLP-1 staining within the ileum of ibuprofen vs. placebo-treated mice (during fasting state). All images are 20× objective lens magnification with 1024×1024 pixels scan format, spanning 100 µm. (**B**) GLP-1 quantification and area in ibuprofen treated mouse and PBS treated; *n* = 3 (PBS), and 4 (Ibp).
